# Impact of COPD and anemia on motor and cognitive performance in the general older population: results from the English longitudinal study of ageing

**DOI:** 10.1186/s12931-020-1305-6

**Published:** 2020-02-03

**Authors:** Inken Padberg, Alice Schneider, Jessica Lee Rohmann, Sean Walter Kelley, Ulrike Grittner, Bob Siegerink

**Affiliations:** 1 Center for Stroke Research Berlin (CSB), Charité Universitätsmedizin Berlin, corporate member of Freie Universität Berlin, Humboldt-Universität zu Berlin, and Berlin Institute of Health, Berlin, Germany; 2 Insititute of Biometry and Clinical Epidemiology, Charité – Universitätsmedizin Berlin, corporate member of Freie Universität Berlin, Humboldt-Universität zu Berlin, and Berlin Institute of Health, Berlin, Germany; 3 Institute of Public Health, Charité – Universitätsmedizin Berlin, corporate member of Freie Universität Berlin, Humboldt-Universität zu Berlin, and Berlin Institute of Health, Berlin, Germany; 4 Department of Psychology, Trinity College Dublin, Dublin, Germany; 5 Berlin Institute of Health (BIH), Berlin, Germany

**Keywords:** Chronic obstructive pulmonary disease, Anemia, Motor function, Cognitive function, Interaction

## Abstract

**Background:**

Cognitive and motor-performance decline with age and the process is accelerated by decline in general health. In this study, we aimed to estimate the effects of COPD and HB levels on cognitive and motor performance in the general older population and assess potential interaction.

**Methods:**

The English Longitudinal Study of Aging is a population-based cohort study including measurements of lung-function and HB levels together with cognitive and motor performance testing. Data were collected from 5709 participants including three measurement time over eight years. COPD was defined using lung-function-parameters and clinical symptoms. HB was assessed continuously and low HB was defined using clinical anemia cutoffs. Linear mixed-effects regression models were used to quantify the associations of COPD and HB with outcome measures, both individually and in combination.

**Results:**

Participants with both low HB and COPD demonstrated worse motor performance compared to individuals with only one exposure, resulting in up to 1 s (95%CI, 0.04–1.8) longer time needed to complete the five times sit to stand task than what would be expected based on purely additive effects. Additionally in individuals with COPD, the time to complete the motor-performance task per unit decrease in continuous HB levels was longer than in participants without COPD after full adjustment for confounding (up to 1.38 s/unit HB level, 95% CI: 0.65–2.11).

**Conclusion:**

In persons with COPD low HB levels may contribute to low motor-performance in a supra additive fashion. Further studies should re-evaluate whether earlier treatment of lower HB in these individuals might be beneficial.

## Background

With increasing age, both cognitive and motor-performance begin to slowly decline [1, 2]. Both functions may influence each other; cognitive impairment has been shown to be associated with an increase in the number of falls among older people, and successfully performing motor tasks, e.g. the five times sit-to-stand test, was found to be inversely associated with cognitive impairment [3, 4].

Next to interdependency, decline of both motor and cognitive function may be further enhanced by certain conditions common in the older population, such as COPD and low HB (hemoglobin) level [5–8]. When individuals suffer from both low HB level and COPD, existing evidence indicates that outcomes such as the development of respiratory failure and mortality in critically ill hospitalized patients but also in the general population may occur more frequently than in persons who suffer only from one condition [9] [10]. Both factors have previously been shown to independently reduce exercise capability and increase feelings of dyspnea [11], while persons suffering from both conditions reported lower quality of life compared to persons with COPD alone [12]. Anemia caused by iron depletion and anemia of chronic disease that occurs in patients with acute or chronic immune activation represent the two most common causes for anaemia [13, 14]. However, especially in an older population also renal insufficiency or vitamin d deficiency may relevant causal factors for low HB levels. Potentially partly associated with chronic inflammation, low HB levels have been reported to occur more frequently among persons with COPD with a prevalence between 10 and 30% in COPD patients and approximately 6% in the general older population [11, 12, 15, 16]. On the other hand, HB levels have been reported to increase by an erythropoietin mediated mechanism that to some degree compensates for the oxygen deprivation characteristic of chronic diseases such as COPD [6].

To our knowledge, no studies thus far have examined in detail how low HB levels alone or in combination with COPD may be associated with cognitive and motor performance in the general older population. In the present study, we aimed to quantify effect estimates for both conditions separately as well as probe whether supra-additive biological effects due to interaction on these performance measures might be present in the general older population.

## Methods

### The ELSA study, main dataset

**Fig. 1:**
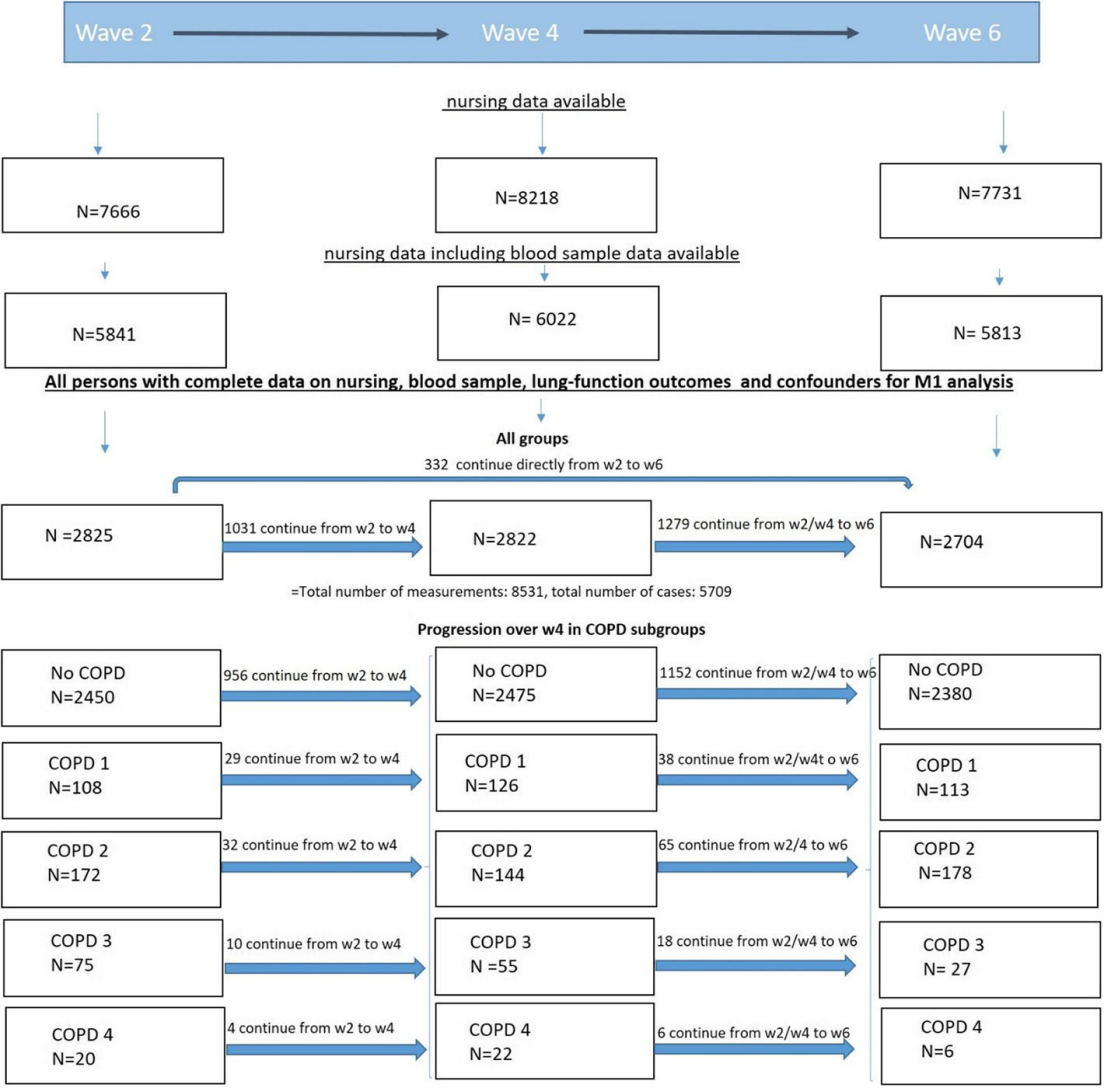
Flowchart. Data used in main model 1 were based on participants for whom blood sample measurements from nursing datasets were available and who also had complete and consistent data on chronic obstructive pulmonary disease (COPD) as well as other variables included in model M1. To maintain the size and representativeness of the panel during waves 3, 4, and 6 additional study participants were recruited. The numbers given in the boxed therefore represent the sum of participants measured for the first time and participants who were already measured at a previous wave. The numbers of participants who continued with complete model 1 data from a previous to next waves are indicated above the arrows connecting the boxes from wave 2 to 4 (w2, w4) and 4 to 6 and for the total number of cases including all COPD subgroups also those that continue from w2 directly to w6. Updated values were used at each measurement time point. The dataset includes 8351 measurements collected over the three waves from 5709 participants

The ELSA (English Longitudinal Study on Ageing) study is a prospective cohort study funded by a consortium of UK-Government departments and the National Institute of Aging in the UK to study the socio-economic and health implications of aging. It collects longitudinal, multidisciplinary data from a representative sample of the English general population aged 50 and older living at private residential addresses [17, 18]. After the first wave of recruitment, 12,099 participants were enrolled and household interviews were conducted. To maintain the size and representativeness of the panel over time, additional study participants aged 50–55 were recruited during waves 3, 4, and 6. In the present study, we used interview data collected every year in combination with the nursing data subset. In this subset of participants, medical information was collected in waves 2, 4 and 6 over a period of 8 years. During wave 2, nursing data were obtained for 7666 participants for the first time and blood samples were collected from 5841 of those participants (Fig. 1).

We excluded participants whose data on the status of lung function and clinical symptoms needed for COPD definition did not match. This means participants who did not report chronic clinical symptoms of COPD (dyspnoea, phlegm, wheezing) but had lung function measurements indicating impairment were excluded. Further excluded were participants fulfilling the definition of ‘healthy’ according to the lung function data but who reported chronic clinical symptoms for COPD (also see measurement section) [19]. Finally, participants with missing data on measurements (15% missing) included in main analysis model (M1) were excluded.

In total, the number of participants with complete household and nursing data that could be used for the main analysis included 2825 participants for wave 2 only (Fig. 1) and 5709 across all waves (age range 50 to 99; collapsed to 99 at 90+ to protect anonymity). From these 5709 participants, a total of 8351 measurements were collected over a maximum of three measurement time points (Fig. 1).

### Measurements

Measurements for the lung function parameters, HB levels, grip strength, blood pressure, motor performance, HBA1C and cholesterol level, used in our analyses were assessed in follow up nurse visits (nursing datasets) wave 2, 4 and 6. Data on clinical symptoms of COPD, age (starting at 50 and older), cognitive performance, alcohol consumption, depression, prior stroke and myocardial infarction (MI) were included as part of the main dataset and measured each wave. The information about prior stroke or MI, clinical symptoms of COPD and alcohol consumption were self-reported during each wave. Number of test reputations was generated by counting the number of waves that the persons continued to participate in the study after baseline assessments.

### Exposures

We defined COPD based on lung function parameters according to GOLD criteria supplemented with information on clinical symptoms. Categorization into healthy (FEV1/FVC > 69% and expected FEV1 > 79), mild (FEV1/FVC < 70%), moderate (FEV1/FVC < 70% and expected FEV1:50–79%), severe (FEV1/FVC < 70% and expected FEV1 30–49%),or very severe (FEV1/FVC < 70% and expected FEV1 < 30) COPD was based on clinical cutoffs based on the severity of airflow restriction as measured by the proportion of expected forced expiratory volume (FEV1) divided by forced vital capacity (FVC) and expected FEV1 [20, 21] (Additional file 1: Table S1). Spirometry measures were obtained without bronchodilator treatment.

Low HB levels were defined using current clinical cut-off levels for anemia (< 12.0 g/dl for women, < 13.5 g/dl for men) [22]. We also assessed HB levels as a mean-centered continuous variable.

### Outcomes

We operationalized functional outcome using both measures of cognitive and motor performance. Cognitive performance (number of words memorized) was measured using delayed words recall with ten words [23]. Motor function (time to complete chair rise) was assessed by measuring the time needed to complete five chair raises. To allow for comparative analysis in the tables and figures, both outcome variables were standardized by dividing mean-centered values by the common standard deviation for each measure. Through this transformation, we could compare the effect estimates for both outcome variables on a common scale. All estimated effects are presented as standardized effect sizes (Cohen’s d). A definition of the supplementary outcomes immediate words recall and balance was included in the supplementary section.

### Confounding variables

We identified the following variables as potential sources of confounding based on a priori knowledge: sex, age, blood pressure, myocardial infarction (MI), stroke, diabetes, total cholesterol, alcohol, smoking, depression and grip strength. Further details on the definition and categorizations used can be found in the supplementary section.

### Statistical analysis

The analysis dataset was generated from the ELSA data repository [17], accessed in May 2018 using SPSS version 22. Subsequent analyses were performed using Stata IC 14.

Baseline characteristics were stratified by COPD function and are reported as median values and interquartile range limits for continuous variables, or absolute numbers and percentages for categorical variables.

To assess the relation between COPD categories and low as well as continuous HB levels (using continuous values measured in mg/dl) on functional outcomes (namely number of words memorized and time to complete chair rises), we used linear mixed-effects regression models. These included individual participants as the level 2 units (random intercept model) and the repeated measurements per individual as the level 1 units. If available, updated variables were incorporated at each new measurement time point. We assessed the relative effects due to (biological) interaction by combining the two categorical exposure variables, COPD and low HB, in six combinations in order to assess whether the combination of both medical conditions results in supra-additive effects with respect to the functional outcomes. The healthy, mild and moderate, as well as the severe and very severe categories of COPD were collapsed into three broader categories of COPD-severity (healthy, mild-to-moderate, and severe-to-very severe, each further divided in a group with and a group without low HB).

In a second analysis a product term between COPD categories and continuous HB levels was introduced to quantify the difference in the effect size of the association between continuous HB levels (in mg/dl) and functional outcome measures in five (healthy, mild, moderate, severe and very severe) different COPD categories.

We used two models to adjust for potential sources of confounding. The first model 1 (M1) included sex and age as well as the number of test repetitions in order to account for the practice effects associated with a better knowledge of the task. In a second fully adjusted model (M2), additional adjustment was made for blood pressure, myocardial infarction (MI), stroke, diabetes, total cholesterol, alcohol, and smoking. Here we also included grip strength and depression according to the CES-D depression scale as markers in M2 to account for the fact that a general decrease in physical or psychological well-being may have also been a source of confounding. We acknowledge grip strength and depression may be intermediates on the causal path, and comment on this in our discussion.

### Handling of missing data

In the fully adjusted analysis, missing data on some of the covariates impacted the number of individuals who could be included in the complete-case analyses. For this reason, missing values for additional confounder variables were imputed based on the complete M1 dataset and available values for M2. Multiple imputation by chained equations (MICE) [24] was conducted to create 10 datasets in which estimates for missing data in the variables alcohol, cholesterol, grip-strength, blood pressure depression, smoking and HBA1C level were calculated using the outcome variables of time needed to complete five chair raises and words recalled, as well as variables without missing values used in M1 namely lung function, HB, age, sex and the number of test repetitions and the non-missing data of the covariates used in M2.

### Sensitivity analysis

Multiple sensitivity analyses were conducted to assess robustness of our findings. This included (a) a complete case analysis of model M2, (b) an analysis incorporating cross-sectional and longitudinal analytical weights for representativeness, and (c) an analysis using a broader more inclusive definition of COPD. Further details on the sensitivity analysis can be found in the supplementary methods section.

## Results

The final dataset used in model M1 included 8351 measurements collected over the three waves from 5709 participants (Fig. 1). Baseline data (meaning first measurements available from each participant, whenever entering the study) from 5709 participants are shown in Table 1.

**Table 1:** Participant baseline characteristics (*n* = 5709 cases)

COPD Status	Healthy, *n* = 4929	Mild *n* = 246	Moderate *n* = 370	Severe *n* = 124	Very severe *n* = 40
Age (years) median [IQR]	61 [56–69]	67 [60–75]	67 [61–73]	68 [63–73]	64 [59–69]
Male sex % (n)	45% (2224)	52% (129)	47% (175)	46% (57)	60% (24)
HB (g/dl) median [IQR]	14.20 [13.40–15.10]	14.20 [13.30–15.10]	14.20 [13.40–15.03]	14.20 [13.40–15.20]	14.55 [13.73–15.40]
Diabetes (as % glycated HB > 6.5) in % (n)	5% (224)	7% (16)	7% (26)	11%(14)	3% (1)
Total cholesterol (mmol/l) median [IQR]	5.80 [5.10–6.60]	5.50[4.80–6.40]	5.50 [4.70–6.30]	5.70 [4.80–6.68]	5.75 [5.00–6.58]
Depression (CES-D scale> = 4) in % (n)	9% (457)	17% (43)	20% (73)	21% (26)	23% (9)
Grip (kg) median [IQR])	29.67 [23.33–40.33]	28.17 [20.67–38.91]	27.67 [21.67–36.00]	26.67 [21.67–32.67]	28.00 [22.75–38.17]
Self-reported prior stroke (yes/no) in % (n)	2% (75)	6% (14)	4% (14)	4% (5)	5% (2)
Self-reported prior MI (yes/no) in% (n)	2% (103)	5% (11)	6% (24)	4% (5)	10% (4)
Self-reported current Smoking in % (n)	7% (405)	15% (37)	30% (109)	36% (44)	33% (13)
Self-reported Alcohol (frequency of days drinking/week) median [IQR]	3 [2–4] *	3 [1–4] *	3 [1–4] *	3 [1–4] *	3[1–4] *
Hypertension^A^ in % (n)	23%*	29% *	34%*	36% *	25% *
Number Words memorized (n) median [IQR]	5.0 [4.0–6.0]	4.50 [3.0–6.0]	5.0 [3.0–6.0]	5.0 [3.0–6.0]	5.0 [4.0–6.0]
Time needed to complete five chair rises (seconds) median [IQR]	10.12 [8.26–12.31]	11.60 [9.06–13.86]	11.62 [9.28–14.57]	13.18 [10.62–15.75]	12.08 [10.15–15.60]

Values represent measured data at baseline without any centering or standardization performed

Abbreviations: *MI* myocardial infarction, *IQR* interquartile range, *HB* hemoglobin, *COPD* chronic obstructive pulmonary disease

* > 5% of data points missing in the following variables: alcohol in healthy (*n* = 3312), mild COPD (*n* = 136), moderate (*n* = 208), severe (*n* = 73), very severe (*n* = 23) and for hypertension in healthy (1075/4629, 23%) mild COPD (66/224, 29%), moderate COPD(116/337,34%),severe COPD (38/107, 36%) and very severe COPD(9/36,25%)

^A^mean systolic at least > 140 or diastolic > 90 or intake of antihypertensive medication in % (n)

Model 2 included additional confounders resulting in 3723 individuals without and 1986 individuals with missing data on additional confounders. The main results presented in Tables 2 and 3 use the dataset with imputation of missing values for additional confounding variables in model M2.

**Table 2:** Effects of the combination of low HB and COPD on cognitive and motor outcomes; standardized effect sizes and 95%CI

COPD category	+low HB	Time to complete chair^a^ rise		Number of Words memorized ^a^	
		M1^b^	M2^c^	M1^a^	M2^c^
NO COPD *N* = 7305^d^	NO *N* = 6770^d^	1 (ref)	1 (ref)	1 (ref)	1 (ref)
	YES = 535^d^	0.14 (0.06 to 0.22)	0.09 (0.02 to 0.17)	−0.14 (− 0.22 to − 0.06)	-0.1 (− 0.18 to 0.02)
Mild and moderate COPD (category 1 and 2) *N* = 841^d^	NO *N* = 735^d^	0.22 (0.15 to 0.29)	0.15 (0.08 to 0.22)	− 0.14 (− 0.21 to − 0.07)	−0.09 (− 0.17 to 0.01)
	YES *N* = 106^d^	0.62 (0.44 to 0.8)	0.5 (0.33 to 0.68)	−0.1 (− 0.28 to 0.08)	−0.01 (− 0.19 to 0.17)
Severe and very severe COPD (category 3 and 4) *N* = 205^d^	NO *N* = 185^d^	0.53 (0.3 to 0.66)	0.41 (0.28 to 0.55)	−0.16 (− 0.3 to − 0.02)	−0.08 (− 0.22 to 0.06)
	YES *n* = 20^d^	0.92 (0.53 to 1.3)	0.8 (0.42 to 1.18)	−0.27 (− 0.66 to 0.12)	− 0.17 (− 0.55 to 0.22)

^a^modeled as the decrease per SD (SD^−1^) in words memorized and time to complete chair raises

^b^adjustment model 1: sex and age, number of test repetitions; *N* = 8351 measurements; 5709 persons

^c^model 2 additionally adjusts for blood pressure, myocardial infarction (MI), stroke, diabetes, total cholesterol, alcohol, depression grip strength and smoking, imputed from complete data for M1 and available values for M2

^d^n = measurements

**Table 3:** Separate effects and effect of the COPD and HB product term on chair rise and word memorization

		Time to complete Chair-rise^b^		Number of words memorized^b^	
		M1 ^d^	M2^e^	M1^d^	M2^e^
COPD^a^	1 vs. 0	0.17 (0.07 to 0.27)	0.11 (0.01 to 0.2)	−0.08 (− 0.18 to 0.02)	−0.04 (− 0.14 to 0.06)
	2 vs. 0	0.31 (0.22 to 0.40)	0.23 (0.14 to 0.32)	−0.15 (− 0.24 to − 0.07)	−0.09 (− 0.18 to − 0.003)
	3 vs. 0	0.58 (0.43 to 0.73)	0.46 (0.31 to − 0.60)	−0.19 (− 0.34 to − 0.03)	−0.10 (− 0.26 to 0.05)
	4 vs. 0	0.74 (0.46 to 1.01)	0.64 (0.37 to 0.91)	−0.12 (− 0.4 to 0.16)	−0.04 (− 0.32 to 0.24)
HB-(g/dl)^c^	Association between lower HB level and time to complete chair rise	0.05 (0.03 to 0.06)	0.034 (0.02 to 0.05)	−0.03 (− 0.04 to − 0.001)	−0.02 (− 0.04 to − 0.001)
COPD + HB (g/dl)	unit decrease in HB *COPD 1 vs. 0	0.1 (0.03 to 0.17)	0.1 (0.04 to 0.17)	0.11 (0.04 to 0.18)	0.11 (0.05 to 0.18)
	Unit decrease in HB*COPD 2 vs. 0	0.04 (−0.02 to-0.11)	0.05 (−0.01 to 0.11)	− 0.02 (− 0.08 to 0.04-)	−0.03 (− 0.09 to 0.03)
	Unit decrease in HB *COPD 3 vs. 0	0.08 (−0.01 to 0.18)	0.11 (0.02 to 0.2)	−0.01(− 0.11 to 0.09)-)	−0.02 (− 0.11 to 0.08)
	Unit decrease in HB *COPD 4 vs. 0	0.36 (0.17 to 0.55)	0.36 (0.17 to 0.54)	−0.03 (− 0.23 to 0.16)	−0.04 (− 0.24 to 0.15)

^a^COPD1 = mild; 2 = moderate; 3 = severe; 4 = very severe

^b^modeled as the decrease per SD (SD^− 1^) in words memorized and time to complete chair raises

^c^HB was included as a continuous variable in g/dl

^d^adjustment model 1: sex and age, number of test repetitions; *N* = 8351 measurements; 5709 persons

^e^model 2 additionally adjusts for blood pressure, myocardial infarction (MI), stroke, diabetes, total cholesterol, alcohol, depression grip strength and smoking, imputed from complete data for M1 and available values for M2

^*^Product term

When looking at baseline data 780 (13.7%) of all participants had some level of COPD at baseline; 616 (10.8%) were mildly or moderately affected (COPD groups 1 + 2) and 164 (2.9%) were affected severely or very severely (COPD groups 3 + 4). Low HB levels (anemia) was present in 7.3% of the healthy participants, in 13.3% of those with mild COPD, in 12.1% of those with moderate COPD, in 11.5% of those with severe and in 4.2% of the participants with very severe COPD. Median HB levels, however, did not differ much between persons with and without COPD (Table 1) but were slightly higher among those with very severe COPD (group 5). As shown in Table 1, among those with COPD, the proportion of males was higher, and the proportion of current smokers was much higher compared to those without COPD. In line with previous findings [25] also depression rates were higher in persons with COPD (Table 1). Furthermore the rates of prior stroke and myocardial infarction (MI) were higher among participants with COPD, and those with COPD were more frequently hypertensive.

In an unadjusted crude comparison of baseline measures, the median time to complete five chair rises was higher in individuals with higher severity of COPD, while the median number of words memorized did not differ substantially upon comparing persons with and without COPD (Table 1). Compared to participants with normal HB levels, the median number of words memorized was lower and the time to complete five chair rises was longer among participants with low HB levels (Additional file 1: Table S2). Correction for analytical weights did not fundamentally alter these crude results (Additional file 1: Table S3).

Finally, in a post hoc analysis assessing unadjusted strata of COPD categories in combination with low versus normal HB levels, CRP-levels were found to be highest among those with severe and very severe COPD and low HB with an average of 14 mg/l compared to 7.2 mg/l in persons with only severe and very severe COPD and 4.4 mg/l in persons with only low HB.

### Individual associations between COPD and low HB on motor and cognitive performance

Having low HB levels was associated with a 0.54 s (95% CI: 0.24–0.84) longer time to complete the motor performance task in model M1. The standardized effect size for low HB level was 0.14 (95% CI: 0.06 to 0.22; Table 2).

In participants with normal HB levels, the presence of mild or moderate COPD was associated with a 0.85 s (95% CI: 0.57–1.14) longer time (standardized effect size: 0.22, 95% CI: 0.15–0.29) to complete the motor performance task in model M1. Those with normal HB levels and severe or very severe COPD needed 2.05 s (95% CI: 1.51–2.58) longer to complete five chair raises (standardized effect size: 0.53 (95% CI: 0.3–0.66)). Associations were attenuated after full adjustment for confounding in M2 (with a standardized effect size of 0.15 (95% CI: 0.08 to 0.22) for mild and moderate and 0.41 (95% CI: 0.28 to 0.55) for severe and very severe COPD). Similar results were obtained in complete case analyses without imputation (Additional file 1: Table S4).

When looking at cognitive performance as measured by the number of words memorized, participants with low HB performed worse: on average − 0.27 (95% CI:-0.42 to − 0.11) fewer words memorized in model M1 (standardized effect size: -0.14 (95% CI: − 0.20 to − 0.06). However, the absolute values of the associations were much smaller after inclusion of additional covariates in M2. Similarly, the effects on the number of words memorized that were observed for mild and moderate COPD (− 0.14(95% CI: − 0.21 to − 0.07)) and for severe and very severe COPD (− 0.16 (95% CI: − 0.3 to − 0.02)) without anemia in model 1 were much smaller after inclusion of additional covariates in model 2.

### Combinations of COPD and low HB levels and their association with motor and cognitive performance

The combination of low HB and the presence of COPD was found to be associated with a lower motor performance than expected based on the purely additive bases. For the chair raises, an additional 1.02 (95% CI: 0.43–1.61) seconds above what would be expected on an additive basis in model M1 were needed for individuals with mild or moderate COPD and an additional 0.98 s (95% CI: 0.15–1.82) for severe and very severe COPD. The corresponding standardized estimates were 0.26 (95%CI 0.11–0.41) and 0.25 (95% CI: 0.04–0.56) (Table 2).

Also after full confounding adjustment, supra-additive effects for the association of COPD and low HB with motor-performance were observed in M2. In participants with low HB, having mild or moderate COPD was associated with a 1.01 s (95% CI: 0.45–1.6, standardized effect size: 0.26 (95% CI: 0.11–0.40)) longer time above of what could be expected by purely additive influences of both exposures individually. Participants with severe or very severe COPD needed 1.15 s ((95% CI: 0.32–1.96), standardized effect size 0.30 (95% CI: 0.08–0.43)) more than what would be expected based on additive effect-estimates.

Results from the sensitivity analyses, namely complete case analysis of M2 (Additional file 1: Table S4) and weight corrected analyses of M1 and M2 shown in Additional file 1: Table S5 were very similar to what was described above.

With regard to the number of words memorized, no supra-additive associations were observed (Table 2).

### Continuous HB levels and motor function among COPD categories

In further support of these results, our analyses including the product term of the continuous HB level and the categorical COPD variable indicated a stronger association between HB levels and motor-performance among participants with COPD. Per unit (g/dl) lower HB level, the time needed to complete the motor performance task was 0.39 s ((95% CI: 0.13–0.65), standardized effect size: 0.10 (95% CI: 0.03 to 0.17)) longer in individuals with mild COPD and 1.39 s ((95% CI: 0.65–2.13), standardized effect size 0.36 (95% CI: 0.17 to 0.55)) longer in participants with very severe COPD (M1 results, Table 3).

**Fig. 2:**
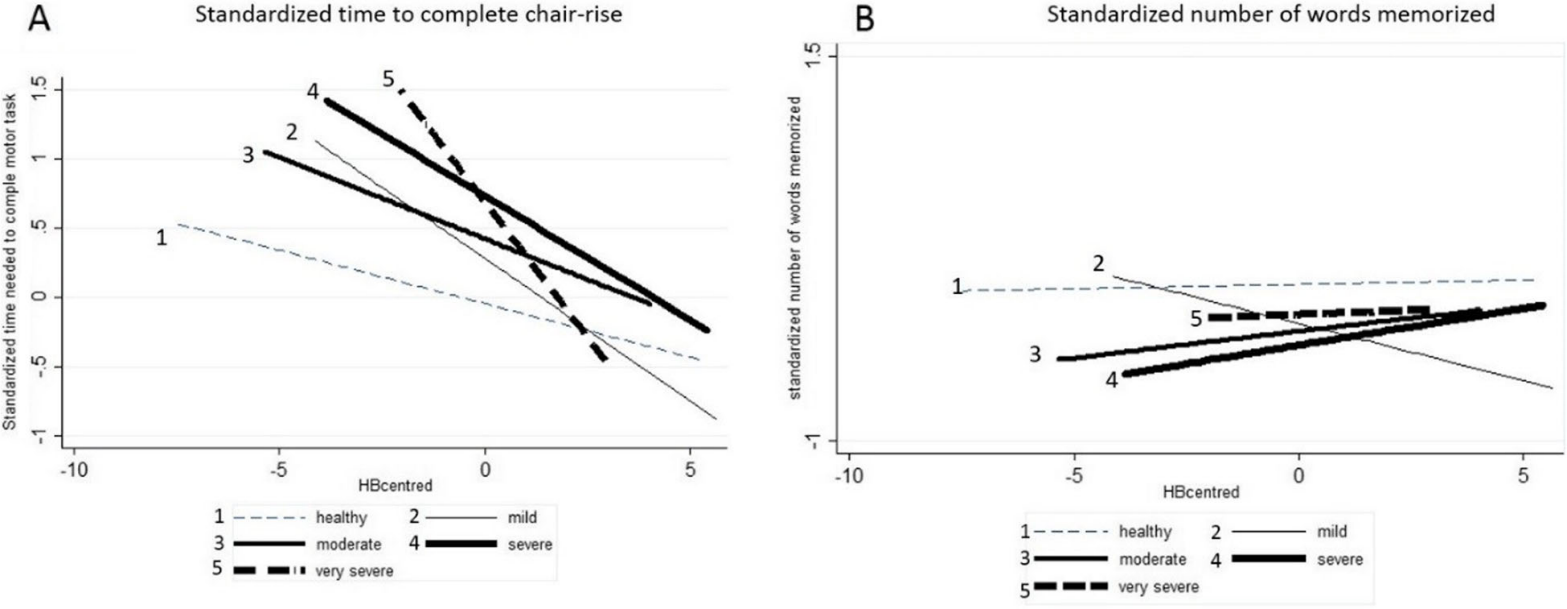
Visualization of the association between HB and outcome measures in participants with different levels of COPD. Depicted is the mean centered HB level against the estimated mean centered time to complete the motor task and the number of words memorized that were normalized by dividing mean cantered values by the standard deviation (**a** and **b**). The association between HB level and cognitive as well as motor-performance is shown for different groups of participants with COPD without confounder correction. **a** Shows that in participants with COPD the time needed to perform the motor task increased more strongly with lower HB levels. In other words, in more severely affected participants with COPD lower HB levels were more strongly associated with a longer time needed to complete the motor-performance task. Overall, the association between a lower HB level and a worse motor function was stronger in participants with severe and very severe COPD (bold dotted and straight lines 4 and 5) than in participants with mild and moderate COPD (lines 2 and 3). **b** Shows the association between HB levels and the number of words memorized. Overall, within most of the COPD groups, with higher levels of HB, slightly more words were memorized on average. However, no major differences between the different COPD groups were seen

The full range of these associations is illustrated in Fig. 2. Here, the association of HB levels and motor or cognitive performance in individuals with different levels of COPD was only corrected for continuous HB levels, COPD and the product term. Figure 2 shows that the slope of the curve describing the association between HB levels and the time needed to complete the motor-performance task was steeper, illustrating the stronger association between HB and motor performance in individuals with compared to those without COPD. In other words if HB levels were higher, on average persons with COPD were more likely to have a motor-performance outcome similar to the average of the healthy persons, or higher HB levels were associated with a better functional outcome especially in persons with COPD (Fig. 2).

The associations observed in M1 remained after additional confounding adjustment but decreased by about 20–30% on average (Table 3). No such associations were observed for the cognitive performance outcome (Table 3).

In sensitivity analyses, similar results were obtained for complete case analysis of M2 (Additional file 1: Table S6) and weight corrected analysis (Additional file 1: Table S7). When using a broader definition for COPD that allowed inclusion of more participants, the observed associations decreased in magnitude (Additional file 1: Table S8).

## Discussion

In our study, we found that both COPD presence and severity and low HB levels were associated with motor performance in the older general population. With regard to cognitive performance no relevant effects were observed. Regarding motor performance, the associations persisted even after correction for confounding factors. Having both low HB levels and COPD was found to have a supra-additive negative effect on the motor but not on the cognitive performance task compared to each exposure alone (Table 2). Furthermore, the association between continuous HB levels and motor performance differed between categories of COPD severity, with moderately stronger positive associations observed between HB levels and motor performance among participants with COPD (Table 3, Fig. 2).

These results are of potentially of high clinical relevance as both COPD and low HB are common in the older population, as was also observed in our study population. Maintaining HB levels might be important for the preservation of motor performance in individuals with COPD and thereby help to facilitate an active lifestyle and reduce following adverse outcomes such as falls and associated mortality.

The observed associations on motor outcomes remained after full confounding adjustment in M2. Here we also included depression and grip strength, which are markers of general psychological as well as physical health, which may confound the relationships under study. However, we acknowledge that these variables may also be mediators of the effects of HB or COPD on the outcome measures, and correction in this case may lead to over-adjustment in these models.

Current HB cutoffs and sex differences in defining anemia might be questionable when used in the older population [20]. HB levels generally decline with age and current studies on HB levels show that especially very high and very low levels in older persons are associated with increased hospitalization and mortality [26]**.** Our results support a re-evaluation of the current cutoffs used to define anemia (low HB) in COPD patients. Defining more specific cut-offs, which may include factors other than sex such as age, has also previously been discussed [22]. Furthermore, HB levels higher than the current cutoffs for anemia have previously been reported to best predict survival in COPD patients [27].

Our results indicate an effect of HB, COPD and their combination on motor-performance but not on the delayed words recall task. This could suggest that the motor effects may be attributable to an acute mismatch between increased oxygen demand and supply during physical activity in participants with COPD and not to a general decline in cognitive motor control. This is supported by the fact that supra-additive effects were also not seen when looking at immediate words recall (Additional file 1: Table S9). We also studied balance, in which cognitive motor control may also be a relevant factor [28]. Only for mild and moderate COPD a trend for small effect was seen in model 1 (standardized effect: 0.15, 95% CI: − 0.01 to 0.32) (Additional file 1: Table S9). This effect however disappeared after full correction in model 2, which further supports our hypothesis that impairment seen in the five times sit to stand test was mainly associated with acute oxygen deprivation during the task and not with a more permanent loss of function in motor control.

However, especially an acute mismatch in oxygen demand and supply that did not yet lead to permanent cognitive damage, might be reversible as the generally most common causes of anemia –iron deficiency and inflammation are potentially treatable. Furthermore also other causes such as for example vitamin D deficiency or renal insufficiency might present plausible underlying causes of anemia that can be treated. Further research on anemia in COPD patients needed to be able to better evaluate treatment options.

### Limitations

Several limitations of our study should be considered. First, our study includes a representative sample of the English population aged 50 and older. Therefore, the median age of our participants at baseline was 61, meaning that many of the participants will first experience stronger effects on cognitive decline later in life. Therefore, results from similar analyses in an even older general populations might reach different conclusions.

Some data were self-reported (e.g. history of alcohol consumption, smoking, dyspnoea, stroke and myocardial infarction) and this may have led to misclassification of these confounding variables [29, 30]. However, the structured, repeated questionnaires were designed to reduce the inaccuracy and reflect uncertainty in participant recall. Furthermore, with regard to cognitive function, more extensive and specific neuropsychological testing would have been preferred. For example, for relational and non-relational material, different memory consolidation rates have been suggested [31]. The test used in the current study may not optimally reflect the capability of our participants to remember facts relevant for their daily living [31]. Despite the longitudinal character of the ELSA study, general attrition of participants high (Fig. 1). General attrition could be caused by participants leaving their private residence and moving into nursing homes. This could lead to an underrepresentation of persons with very severe COPD, as they might be too severely impaired to live at home and participate in the ELSA study. However, the results from analyses of a subset of participants for whom analytical weights were given in an attempt to make the study population more representative of the general UK population, led to similar results as our main analyses.

## Conclusion

We set out to study the effect of HB levels and COPD, both individually and in combination, on cognitive and motor-performance in the older population. These relationships had yet to be evaluated in a population-based setting. Our findings indicate that while cognitive performance as measured by a recall task does not seem to be affected, both lower HB levels and more severe COPD were each associated with a worse motor-performance in an older general population.

Moreover, individuals with low HB and COPD showed a lower motor-performance than what would be expected based on the additive individual effects alone and motor functional outcomes were more strongly dependent on HB levels in individuals with COPD. Future studies should investigate in more detail by which mechanism and at which precise cutoff lower HB levels in combination with COPD may cause a supra-additive decline in motor-performance in older persons. We also recommend a formal evaluation and optimization of current HB cutoff levels, and assessment of whether earlier treatment of low HB might prevent falls and associated morbidity and mortality in the older population.

## Additional file

**Additional file 1:** **Table S1.** COPD categorization based on pulmonary function. **Table S2.** Anemia vs. outcome measures at baseline. **Table S3.** Weight corrected participant baseline characteristics. **Table S4.** Complete case analysis of model M2: Effects of the combination of low HB and COPD on cognitive and motor outcomes. **Table S5.** Effects of the combination of low HB and COPD on cognitive and motor outcomes (with and without correction for cross-sectional and longitudinal weight including only the subgroup of participants for whom longitudinal weights were available). **Table S6.** Complete case analysis of M2, Separate effects and effect of the COPD and HB product term on chair rise and word memorization. **Table S7.** Separate and effect of the COPD and HB product term on chair rise (with and without correction for analytical weigths, including only the subgroup of participants for whom multiple measures and longitudinal weights were available). **Table S8.** Separate effects and effect of the COPD and HB product term on chair rise and word memorization without clinical parameters. **Table S9.** Effects of the combination of low HB and COPD on immediate words recall and balance.

## Data Availability

The data are publicly available through the UK data archive. ELSA was developed, and data were collected, by a team based at the NatCen social research, University College London and the Institute for fiscal studies. Data are available via the UK-Data repository: https://beta.ukdataservice.ac.uk/datacatalogue/series/series?id=200011#!/access-data

## Abbreviations

COPD: Chronic Obstructive Pulmonary Disease
FEV: Forced expiratory volume
HB: Hemoglobin
IQR: Interquartile range
MI: Myocardial infarction
