# Supplementary material for: Impact of COPD and anemia on motor and cognitive performance in the general older population: results from the English longitudinal study of ageing
Source: Respir Res. 2020 Feb 3;21:40. doi: 10.1186/s12931-020-1305-6 (PMC6998076; doi:10.1186/s12931-020-1305-6)
Supplement: Supplementary file 1 — Additional file 1: Table S1. COPD categorization based on pulmonary function. Table S2. Anemia vs. outcome measures at baseline. Table S3. Weight corrected participant baseline characteristics. Table S4. Complete case analysis of model M2: Effects of the combination of low HB and COPD on cognitive and motor outcomes. Table S5. Effects of the combination of low HB and COPD on cognitive and motor outcomes (with and without correction for cross-sectional and longitudinal weight including only the subgroup of participants for whom longitudinal weights were available). Table S6. Complete case analysis of M2, Separate effects and effect of the COPD and HB product term on chair rise and word memorization. Table S7. Separate and effect of the COPD and HB product term on chair rise (with and without correction for analytical weigths, including only the subgroup of participants for whom multiple measures and longitudinal weights were available). Table S8. Separate effects and effect of the COPD and HB product term on chair rise and word memorization without clinical parameters. Table S9. Effects of the combination of low HB and COPD on immediate words recall and balance. [file 12931_2020_1305_MOESM1_ESM.docx]

**Supplementary material and methods, results and discussion**

**Methods**

Sensitivity analyses

We used linear mixed-effects regression models including individual participants as the level 2 units (random intercept model) with the repeated measurements per individual as the level 1 units for all sensitivity analysis. If available, updated variables were used at each new measurement time point

In addition to the main analysis of model 2 including imputed values for missing additional confounding variables used in M2 a complete case analysis without imputation for M2, including data from 3,723 persons across all waves, was done.

A second sensitivity analysis was performed in which results of the multilevel model were corrected for cross-sectional as well as longitudinal (level 2) analytical weights.

Level 1 measurements were corrected for cross-sectional weights that were generated by the ELSA studies for all participants who had their blood samples taken during nurse visits. Additional correction for longitudinal weights was done on the individual level (level 2) when individuals were contributing data at multiple waves. Longitudinal weights were only available for persons with multiple measurement time points who took part in the main interviews in all waves, up to and including the last wave included. For persons who missed one main interview but returned to the study at a later wave, or for those who joined the study at a later wave, no longitudinal weights were generated. As a consequence, performing weight correction on our two-level model was only possible for 4,180 participants [^1^](#_ENREF_1). Analysis was done in Stata with control scaling of sampling weights.

In a third sensitivity analysis, we probed whether our results were robust to additionally including participants who only met the definition of COPD based on only lung function measurements but not on clinical symptoms. This resulted in a larger dataset (N=7,667).

All data from the ELSA study are open-access and can be freely downloaded from the UK Databank [^1^](#_ENREF_1).

Definition of confounding variables

Information about participant sex was dichotomous. Age was included as a continuous variable and the number of test repetitions as an ordinally in the relevant models. Furthermore hypertension was defined as having a mean systolic blood pressure of at least≥ 140 mmHg or diastolic blood pressure ≥ 90 mmHg over three measurements taken on the right arm with a sphygmomanometer, or based on self-reported anti-hypertensive medication use. Diabetes was categorized using HBA1C cut-offs (healthy: percent glycated HB (HBA1C) < 5.7, pre-diabetic: HBA1C ≥ 5.7 and <6.5 and diabetic: HBA1C≥6.5). Total cholesterol was treated as continuous variables measured in mmol/L. The number of previous myocardial infarctions and strokes, if any, were included as ordinal variables. Alcohol consumption was grouped by frequency in the last 12 months (1= almost every day, 2 five or six days a week, 3= three or four days a week, 4= once or twice a week, 5=once or twice a month, 6= once every couple of months, 7=once or twice a year, 8= not at all). Smoking behavior was classified as current smoking, ever smoking or never smoking. Finally depression information was classified in three categories based on the CES-D depression scale (no depression (0), mild depression (1-3), depression (4-8)) and included as an ordinal variable and grip strength continuously as measured using a Jama-dynamometer [^2^](#_ENREF_2). CRP was measured in mg/L.

Supplementary outcomes:

Immediate words recall: Similar to delayed words recall immediate words recall was tested using 10 words. In the immediate words recall test the subjects had to repeat these words immediately after they were presented.

Balance: Included were all persons who attempted the semi and full tandem stand as part of the nursing dataset. Respondents who completed the semi tandem stand for 10 seconds were then asked to do the full tandem stand. If the respondent was aged 69 and under they were asked to attempt the full tandem stand for 30 seconds; if they were 70 or over they were asked to do the full tandem stand for 10 seconds. The outcome of balance was generated by combining the outcomes from the semi and the full tandem stand as follows: Points were given depending on the combined performance in the two tests. If neither the semi nor the full tandem stand was attempted 5 points were given, if the semi tandem stand was held for less than 10 seconds 4 points were given, if persons did not attempt the full tandem stand but had completed the semi tandem stand 3 points were given, if persons could held the semi tandem stand for the full ten seconds but the full tandem stand for less than the required amount of time two points were given and if persons completed both test and could held the position for the required amount of time, 1 point was given.

**Supplementary Results**

**Supplementary table 1**: COPD categorization based on pulmonary function

| category | FEV1/FVC<70% | Expected FEV1  50-79 | Expected FEV 30-49% | Expected FEV<30% |
| --- | --- | --- | --- | --- |
| COPD0 (healthy) | No | No | No | no |
| COPD1 (mild) | Yes | no | no | no |
| COPD2 (moderate) | Yes | yes | no | no |
| COPD3 (severe) | Yes | no | yes | no |
| COPD4 (very severe) | Yes | no | No | yes |

For the main analysis data on pulmonary function furthermore were combined with self-reported chronical clinical symptoms (dyspnoe/phlegm/wheezing) to be classified as COPD patients, while for the definition of healthy patients reporting chronic clinical symptoms were excluded. For a secondary analysis.

FEV= forced expiratory volume

**Supplementary table 2**

Table 2: Anemia vs. outcome measures at baseline

| Anemia, HB<12.0 g/dl for women or <13.5g/dl for men | no | yes |
| --- | --- | --- |
| Number Words memorized ,n [median, IQR] | 5 [4-6] | 4 [3-6] |
| Time needed to complete five chair rises (seconds)[median, IQR] | 10.28 [8.34-12.46] | 11.43 [9.04-15.08] |

IQR=inter quartile range

**Supplementary table 3:** Weight corrected participant baseline characteristics

| COPD Status | Healthy, n=5044 | Mild  n=242 | Moderate n=371 | Severe n=130 | Very severe n=44 |
| --- | --- | --- | --- | --- | --- |
| Age (years) median [IQR] | 59 [54-89] | 67 [60-75] | 67 [60-73] | 68 [63-74] | 62 [57-69] |
| Male sex % (n) | 47% (2392) | 51% (123) | 49% (183) | 49% (64) | 59% (26) |
| HB (g/dl) median [IQR] | 14.2 [13.40-15.10] | 14.20 [13.24-15.10] | 14.20 [13.50-15.1] | 14.19 [13.30-15.20] | 14.58 [13.75-15.40] |
| Diabetes (as % glycated HB >6.5) in % (n) | 5% (231) | 8% (19) | 7% (27) | 12%( 16) | 2% (1) |
| Total cholesterol (mmol/l) median [IQR] | 5.80 [5.10-6.60] | 5.50 [4.80-6.40] | 5.50 [4.60-6.30] | 5.58 [4.70-6.51] | 5.56 [4.87-6.57] |
| Depression (CES-D scale>=4) in % (n) | 10% (521) | 21% (52) | 20% (75) | 24% (31) | 27% (12) |
| Grip (kg) median [IQR]) | 30.00 [23.33-41.00] | 27.36 [19.33-37.67] | 28.00  [21.67-36.11] | 27.00 [21.45-33.33] | 27.79 [22.67-37.73] |
| Self-reported prior stroke (yes/no) in % (n) | 1% (70) | 5% (13) | 4% (16) | 5% (6) | 5% (2) |
| Self-reported prior MI (yes/no) in% (n) | 2% (95) | 5% (13) | 7% (26) | 5% (6) | 11% (5) |
| Self-reported current Smoking in % (n) | 10% (495) | 17% (41) | 33% (121) | 40% (51) | 36% (16) |
| Self-reported Alcohol (frequency of days drinking/week) median [IQR] | 3 [2-4] * | 3 [1-4] * | 3 [1-4] * | 3 [1-4] * | 3[1-4] * |
| Hypertension in % (n) | 23%* | 30% * | 36%* | 36% * | 25% * |
| Number Words memorized (n) median[IQR] | 5.0 [4.0-6.0] | 4.00 [3.0-6.0] | 4.0 [3.0-5.0] | 5.0 [3.0-6.0] | 5.0 [4.0-6.0] |
| Time needed to complete five chair rises (seconds) median [IQR] | 10.01 [8.20-12.22] | 11.95 [9.29-14.93] | 11.67 [9.43-14.68] | 13.46 [10.60-15.75] | 12.16 [10.25-15.87] |

Values represent measured data at baseline without any centering or standardization performed.

Abbreviations: MI=myocardial infarction, IQR=interquartile range, HB= hemoglobin, COPD= chronic obstructive pulmonary disease

*> 5% of data points missing in the following variables: alcohol in healthy (n=3276), mild COPD (n=126), moderate (n=199), severe (n=74), very severe (n=24) and for hypertension in healthy (1083/4723, 23%) mild COPD (66/219, 30%), moderate COPD(119/334,36%),severe COPD (39/110, 36%) and very severe COPD (10/39,25 %).

^A^ mean systolic at least > 140 or diastolic > 90 or intake of antihypertensive medication in % (n)

**Supplementary table 4:** Complete case analysis of model M2: Effects of the combination of low HB and COPD on cognitive and motor outcomes

|  |  | Model M2^1^ | |
| --- | --- | --- | --- |
| COPD category | + low HB | Chair rise^2^ | Words memorized^2^ |
| NO COPD | NO | 1 (ref) | 1 (ref) |
|  | YES | 0.09 (-0.003 to 0.18) | -0.06 (-0.16 to 0.05) |
| Mild and moderate COPD (category 1 and 2) | NO | 0.12 (0.03 to 0.21) | -0.1 (-0.2 to -0.003) |
|  | YES | 0.55 (0.31 to 0.77) | -0.02 (-0.28 to 0.23) |
| Severe and very severe COPD (category 3 and 4) | NO | 0.44 (0.26 to 0.62) | 0.02 (-0.17 to 0.21) |
|  | YES | 0.68 (0.13 to 1.23) | -0.30 (-0.9 to 0.30) |

1: Results are normalized by the standard deviation (SD^-1^)

2: model M2 complete case; N=5083 measurements; 3723 persons

**Supplementary table 5**: Effects of the combination of low HB and COPD on cognitive and motor outcomes (with and without correction for cross-sectional and longitudinal weight including only the subgroup of participants for whom longitudinal weights were available).

|  |  | Time to complete chair rises ^1^ | | Time to complete chair rises ^2^ | |
| --- | --- | --- | --- | --- | --- |
| COPD category | +low HB | M1^3^ | M2^4^ | M1^3^ | M2^4^ |
| NO COPD | NO | 1 (ref) | 1 (ref) | 1 (ref) | 1 (ref) |
|  | YES | 013(0.03-0.23) | 0.07(-0.04-0.17) | 0.14(0.06-0.23) | 0.10(0.01-0.18) |
| Mild and moderate COPD (category 1 and 2) | NO | 0.16(0.06-0.26) | 0.1(-0.001-0.2) | 0.16(0.08-0.24) | 0.11(0.02-0.19) |
|  | YES | 0.63(0.24-1.02) | 0.51(0.13-0.89) | 0.53(0.34-0.72) | 0.43(0.24-0.62) |
| Severe and very severe COPD (category 3 and 4) | NO | 0.45(0.28-0.62) | 0.34(0.17-0.51) | 0.47(0.32-0.62) | 0.37(0.22-0.52) |
|  | YES | 0.77(0.06-1.48) | 0.65(-0.06-1.37) | 0.64 (0.24-1.04) | 0.56(0.16-0.95) |

1: with correction for cross-sectional and longitudinal weight, results are normalized by the standard deviation (SD^-1^)

2: without correction for cross-sectional and longitudinal weight, results are normalized by the standard deviation (SD^-1^)

3: adjustment model M1: sex and age, number of test repetitions; N=6252 measurements; 4180 persons

4: model M2 additionally adjusts for blood pressure, myocardial infarction (MI), stroke, diabetes, total cholesterol, alcohol, depression grip strength and smoking, imputed from complete data for M1 and available values for M2

**Supplementary table 6:** Complete case analysis of M2, Separate effects and effect of the COPD and HB product term on chair rise and word memorization

|  |  | Time to complete Chair-rise^1^ |  | Number of words memorized^1^ |
| --- | --- | --- | --- | --- |
|  |  | M2 ^2^ |  | M2 ^2^ |
| COPD^3^ | 1 vs. 0 | 0.11 (-0.01 to 0.24) |  | -0.08 (-0.21 to 0.06) |
|  | 2 vs. 0 | 0.2 (0.09 to 0.31) |  | -0.09 (-0.21 to 0.03) |
|  | 3 vs. 0 | 0.58 (0.38 to 0.79) |  | 0.03 (-0.19 to 0.25) |
|  | 4 vs. 0 | 0.41 (0.09 to 0.73) |  | -0.14 (-0.5 to 0.22) |
|  |  |  |  |  |
| HB (g/dl)^4^ | Association between lower HB-level and time to complete chair rise | 0.04 (0.02 to 0.06) |  | -0.01 (-0.04 to 0.01) |
|  |  |  |  |  |
| COPD + HB-level (g/dl) | Unit decrease in HB-level *COPD 1 vs. 0 | 0.02 (-0.07 to 0.11) |  | 0.12 (0.02 to 0.22) |
|  | Unit decrease in HB-level *COPD 2 vs. 0 | 0.1 (0.03 to 0.17) |  | -0.02 (-0.10 to 0.06) |
|  | Unit decrease in HB-level *COPD 3 vs. 0 | 0.16 (0.02 to 0.3) |  | -0.03 (-0.19 to 0.13) |
|  | Unit decrease in HB-level *COPD 4 vs. 0 | 0.36 (0.11 to 0.61) |  | -0.09 (-0.36 to 0.19) |

1: Results are normalized by the standard deviation (SD^-1^)

2: model M2 complete data available, N=5083 measurements; 3723 persons

3: COPD1 =mild; 2=moderate; 3=severe; 4=very severe

4: HB was included as a continuous variable in g/dl

**Supplementary table 7**: Separate and effect of the COPD and HB product term on chair rise (with and without correction for analytical weigths, including only the subgroup of participants for whom multiple measures and longitudinal weights were available).

|  |  | Time to complete chair rises ^1^ | | Time to complete chair rises ^2^ | |
| --- | --- | --- | --- | --- | --- |
|  |  | M1^3^ | M2^4^ | M1^3^ | M2^4^ |
|  |  |  |  |  |  |
| COPD + HB (g/dl) | Unit decrease in HB-level *COPD 1 vs. 0 | 0.14 (0.03-0.25) | 0.14 (0.03-0.25) | 0.1 (-0.03-0.67) | 0.01 (0.03-0.17) |
|  | Unit decrease in HB-level *COPD 2 vs. 0 | 0.06 (-0.04-0.16) | 0.06 (-0.04-0.17) | 0.08 (0.01-0.15) | 0.08 (0.01-0.15) |
|  | Unit decrease in HB-level *COPD 3 vs. 0 | 0.09 (-0.06-0.25) | 0.1 (-0.05-0.25) | 0.07 (-0.03-0.17) | 0.09 (-0.05-0.19) |
|  | Unit decrease in HB-level *COPD 4 vs. 0 | 0.36 (0.09-0.63) | 0.35 (0.07-0.62) | 0.32 (0.11-0.52) | 0.33 (0.12-0.53) |

1: with correction for cross-sectional and longitudinal weight, results are normalized by the standard deviation (SD^-1^)

2: without correction for cross-sectional and longitudinal weight, results are normalized by the standard deviation (SD^-1^)

3: adjustment model M1: sex and age, number of test repetitions; N=6252 measurements; 4180 persons

4: model M2 additionally adjusts for blood pressure, myocardial infarction (MI), stroke, diabetes, total cholesterol, alcohol, depression grip strength and smoking, imputed from complete data for M1 and available values for M2

**Supplementary Table 8:** Separate effects and effect of the COPD and HB product term on chair rise and word memorization without clinical parameters

|  | Chair rise ^2^ | Chair rise ^2^ | Words memorized^2^ | Words memorized^2^ |
| --- | --- | --- | --- | --- |
|  | M1^4^ | M2^5^ | M1^4^ | M2^5^ |
| COPD^1^ 1 vs. 0 | -0.06 (-0.10 to -0.02) | -0.06 (-0.11 to -0.01) | -0.02 (-0.06 to0.03) | -0.03 (-0.09 to 0.02) |
| COPD^1^ 2 vs. 0 | 0.15 (0.1 to 0.2) | 0.09 (0.03 to 0.15) | -0.08 (-0.14 to -0.03) | -0.04 (-0.11 to 0.03) |
| COPD^1^ 3 vs. 0 | 0.27 (0.17 to 0.36) | 0.3 (0.18 to 0.41) | -0.09 (-0.18 to 0.01) | 0.01 (-0.11 to 0.14) |
| COPD^1^ 4 vs. 0 | 0.19 (0.04-0.33) | 0.16 (-0.02 to 0.33) | -0.04 (-0.19 to 0.1) | -0.2 (-0.39 to -0.01) |
|  |  |  |  |  |
| Association between lower HB^3^ level and time to complete chair rise | 0.04 (0.02 to 0.06) | 0.04 (0.0-to 0.06) | -0.03 (-0.04 to 0.01) | -0.01 (-0.03 to 0.02) |
| Unit decrease in HB*COPD 1 vs. 0 | 0.02 (-0.01 to 0.06) | 0.01 (-0.05 to 0.03) | -0.04 (-0.07 to 0.01) | -0.03 (-0.08 to 0.01) |
| Unit decrease in HB *COPD 2 vs. 0 | 0.02 (-0.01 to 0.06) | 0.03 (-0.01 to 0.08 ) | 0.001 (-0.04 to 0.04 ) | -0.01 (0.06 to 0.04 ) |
| Unit decrease in HB *COPD 3 vs. 0 | 0.03 (-0.04 to 0.1) | 0.06 (-0.03 to 0.15) | -0.02 (-0.08 to 0.05) | -0.004(-0.1 to 0.09) |
| Unit decrease in HB *COPD 4 vs. 0 | 0.09 (-0.01 to 0.19) | 0.14 (0.02 to 0.26) | 0.03(-0.07 to 0.14) | -0.02(-0.15 to 0.12) |

1: COPD1 =mild; 2=moderate; 3=severe; 4=very severe; all data available for patients with COPD according to lung function measurements are presented without taking into account the presence of clinical symptoms.

2: modeled as the decrease per SD (SD^-1^) in words memorized and time to complete chair raises

3: HB was included as a continuous variable in g/dl

4: adjustment model M1: sex and age, number of test repetitions: N=12474 measurements, 7667 persons

5: model M2 additionally adjusts for blood pressure, myocardial infarction (MI), stroke, diabetes, total cholesterol, alcohol, depression grip strength and smoking, imputed from complete data for M1 and available values for M2

Table 2: Effects of the combination of low HB and COPD on cognitive and motor outcomes; standardized effect sizes and 95%CI

**Supplementary Table 9:** Effects of the combination of low HB and COPD on immediate words recall and balance

|  | balance | Number of Words memorized ^1^ (immediate recall) |
| --- | --- | --- |
| COPD category | M1^,2^ | M1^1^ |
| NO COPD | 1 (ref) | 1 (ref) |
|  | 0.14 (0.05-0.23) | -0.11(-0.2 to -0.03) |
| Mild and moderate COPD (category 1 and 2) | 0.16 (0.08 to 0.23) | -0.14  (-0.21 to -0.06) |
|  | 0.45 (0.26 to 0.64) | -0.25 (-0.43 to 0.07) |
| Severe and very severe COPD (category 3 and 4) | 0.31(0.17 to 0.46) | -0.15(-0.29 to -0.01) |
|  | 0.27 (-0.15 to 0.69) | -0.43 (-0.66 to 0.39) |

1: modeled as the decrease per SD (SD^-1^) in words memorized and time to complete chair raises

2: adjustment model 1: sex and age, number of test repetitions; N=8351 measurements; 5709 persons

**Supplementary discussion**

Unadjusted median HB-levels in our study (Table 1) were found to be higher among patients with very severe COPD than in healthy participants without COPD. The latter could be associated with an erythropoetin-mediated mechanism that may naturally help patients with severe COPD to compensate for the decreased oxygen availability [^3^](#_ENREF_3). However we also observed an overall higher percentage of anemia in COPD compared to healthy participants. This difference could partly be explained by the fact that inflammatory processes may also cause a decrease of HB levels especially in some of the patients with COPD. We can further attribute the discrepancy to the fact that the percentage of males on average was higher in persons with COPD. Current cutoffs used to define anemia are less strict for males. Thus more males and therefore an average also more COPD patients fulfilled the criteria for anemia. The current definition for anemia cutoffs maybe more meaningful in younger patients as ten years after menopause general HB levels in woman have been shown to not differ much from those in man [^4^](#_ENREF_4).

Low ferritin levels can be an indicator of anemia caused by iron deficiency. However ferretin-levels are also found to be increased in association with inflammation in chronic disease ^[5](#_ENREF_5),[6](#_ENREF_6)^ potentially masking iron deficiency in these cases. In line with this also in our study the levels of ferretin were found to be higher in severe and very severe COPD (143 ng/ml) and lower in anemia (107ng/ml) as it was to be expected. In groups combining anemia and COPD, ferritin levels were similar to healthy controls (130 ng/ml in severe and very severe COPD plus anemia and 134ng/ml in healthy participants) which could indicate that the two effects leading to a decrease or an increase in ferritin levels were balancing each other out.

The overall association observed for HB/COPD and well as their interactions were much stronger when defining COPD by lung function and clinical symptoms than when it was defined by lung function alone even though case numbers increased with the less strict definition of COPD (Supplementary table 8). We believe the less precise definition of COPD may have introduced some misclassification of the exposure and would interpret those results with caution.

**References**

1. Research NS. English Longitudinal Study of Ageing. In.

2. Peters MJH, van Nes SI, Vanhoutte EK, et al. Revised normative values for grip strength with the Jamar dynamometer. *Journal of the Peripheral Nervous System.* 2011;16(1):47-50.

3. Kent BD, Mitchell PD, McNicholas WT. Hypoxemia in patients with COPD: cause, effects, and disease progression. *International Journal of Chronic Obstructive Pulmonary Disease.* 2011;6:199-208.

4. Rushton DH, Dover R, Sainsbury AW, Norris MJ, Gilkes JJ, Ramsay ID. Why should women have lower reference limits for haemoglobin and ferritin concentrations than men? *BMJ (Clinical research ed).* 2001;322(7298):1355-1357.

5. DOUGLAS L. SMITH. *Am Fam Physician.* 2000:1565-1572.

6. Weiss G, Ganz T, Goodnough LT. Anemia of inflammation. *Blood.* 2019;133(1):40-50.
